# Low risk of haematomas with intramuscular vaccines in anticoagulated patients: a systematic review with meta-analysis

**DOI:** 10.1186/s12959-022-00367-1

**Published:** 2022-02-16

**Authors:** Daniel Caldeira, Bárbara Sucena Rodrigues, Mariana Alves, Fausto J. Pinto, Joaquim J. Ferreira

**Affiliations:** 1 Laboratory of Clinical Pharmacology and Therapeutics, Faculdade de Medicina, Universidade de Lisboa, Lisboa, Portugal; 2 Centro Cardiovascular da Universidade de Lisboa - CCUL, CAML, Faculdade de Medicina, Universidade de Lisboa, Lisboa, Portugal; 3 Cardiology Department, Hospital Universitário de Santa Maria – CHULN, Santa Maria, Portugal; 4 Faculdade de Medicina da Universidade de Lisboa, Lisboa, Portugal; 5 Instituto de Medicina Molecular João Lobo Antunes, Faculdade de Medicina, Universidade de Lisboa, Lisboa, Portugal; 6 Serviço de Medicina III, Hospital Pulido Valente, CHLN, Lisboa, Portugal; 7 CNS - Campus Neurológico Sénior, Torres Vedras, Portugal; 8 Laboratório de Farmacologia Clínica e Terapêutica - CCUL, Faculdade de Medicina, Universidade de Lisboa, Av. Prof. Egas Moniz, 1649-028 Lisboa, Portugal

**Keywords:** Bleeding, Haemorrhage, Vaccine, Flu, anticoagulation

## Abstract

**Introduction:**

The summary of product characteristics of vaccines administered intramuscularly, including the vaccine for coronavirus SARS-CoV-2 (COVID-19) and Influenza, warned for risks of bleeding in patients treated with oral anticoagulants. We aimed to estimate the incidence of major bleeding events in this setting and to compare these risks against other vaccination routes.

**Methods:**

This systematic review included all prospective and retrospective studies enrolling anticoagulated patients that received intramuscular vaccination, published until December 2020 in CENTRAL, MEDLINE and EMBASE. The outcomes of interest were major bleeding and haematoma related with vaccination. The incidence of the outcomes was estimated through a random-effects meta-analysis using the Freeman-Turkey transformation. The results are expressed in percentages, with 95%-confidence intervals (95%CI), limited between 0 and 100%. When studies compared intramuscular vaccination vs. other route, the data were compared and pooled using random-effects meta-analysis. Risk ratios (RR) with 95%CI were reported.

**Results:**

Overall 16 studies with 642 patients were included. No major bleeding event was reported. The pooled incidence of haematomas following vaccination (mostly against Influenza) in patients treated with oral anticoagulants (mostly warfarin; no data with DOACs / NOACs) was 0.46% (95%CI 0-1.53%). Three studies evaluated the intramuscular vs. subcutaneous route of vaccination. Intramuscular vaccines did not increase the risk of haematoma (RR 0.53, 95%CI 0.10-2.82) compared with subcutaneous route.

**Conclusions:**

Intramuscular vaccination in anticoagulated patients is safe with very low incidence of haematomas and the best available evidence suggests that using the intramuscular route does not increase the risk of haematomas compared with the subcutaneous route.

**Supplementary Information:**

The online version contains supplementary material available at 10.1186/s12959-022-00367-1.

## Introduction

Oral anticoagulants are used to treat or prevent thromboembolic events. Atrial fibrillation, venous thromboembolism, and mechanical prosthesis are the main indications for the use of these drugs. Despite the invasiveness of intramuscular injections, oral anticoagulation is not discontinued in vaccination contrary to what occurs before major surgeries due to the increased risk of bleeding.[1, 2] Nevertheless, the summary of product characteristics of vaccines administered intramuscularly recommend precaution in patients with coagulation disorders, due to potential risk of bleeding after intramuscular injection.[3] This issue has raised some doubts, particularly for the recent vaccine against coronavirus SARS-CoV-2 (COVID-19).[4].

To further elucidate all stakeholders about the risks of intramuscular vaccines in anticoagulated patients, we aimed to perform a systematic review to estimate the incidence of hemorrhagic complications in this setting and to compare the hemorrhagic risks of intramuscular vaccination against other routes, namely subcutaneous.

## Methods

This systematic review has been developed based on the applicable aspects of Preferred Reporting Items for Systematic review and Meta-Analysis (PRISMA) guidelines and Meta-Analysis Of Observational Studies in Epidemiology (MOOSE) Checklist.[5, 6].

### Types of studies included

This systematic review aimed to enrol all interventional or observational studies, including randomized controlled trials, quasi-randomized clinical trials, cohort/nested case-control studies, case-control studies, either prospective or retrospective. Studies had to include at least one arm with anticoagulated patients receiving vaccines deemed to be administrated through intramuscular route. For eligibility we considered all types of oral anticoagulation, i.e. vitamin K antagonists (warfarin, acenocoumarol, phenprocoumon, fluindione) or direct oral anticoagulants (DOACs / NOACs: dabigatran, apixaban, edoxaban, rivaroxaban) or oral anticoagulation without specifying the used drugs. Case reports and case series of bleeding events were excluded.

### Types of outcome measures

The primary outcomes were: (1) the incidence of major bleeding events; (2) the incidence of local haematoma. The secondary outcome was the increase of arm circumference as a surrogate of local complication, defined as an increase of at least 1 cm or swollen arm as defined by the investigators.

### Search methods for identification of studies

We searched for studies in the following electronic databases: Cochrane Central Register of Controlled Trials (CENTRAL), MEDLINE, EMBASE; from inception until 18th December 2020. The full search strategy is presented in the Table 1 of the Supplementary Data Appendix.

### Data extraction and risk of bias evaluation

Two reviewers (BSR and MA) screened the titles and abstracts yielded by the searches against the inclusion criteria. In a second phase, the full text reports were assessed independently by the reviewers to determine whether these met the inclusion criteria. Disagreements were solved by consensus or recurring to a third party (DC). The reasons for exclusion at this stage were recorded and are detailed in Table 2 of the Supplementary Data Appendix.

The data from the individual studies identified for inclusion was introduced into a pre-piloted form. This information included: authors, year of publication; sample size; participants’ characteristics; anticoagulant used; indication for oral anticoagulation; vaccines used, measures before and after vaccination.

The risk of bias evaluation of the included studies was performed using a scale adapted from Hoy and colleagues[7, 8]. This tool evaluates the representativeness of the sample, the sampling technique, the response rate, the data collection method, the measurement tools, the case definitions, and the statistical reporting. According to this score the risk of bias of the studies were categorised as “low risk” (7-9 points), “moderate risk” (4-6 points), or “high risk” (0-3 points).[8] For randomized controlled trial evaluating the intramuscular route against others, the Cochrane Risk of Bias Tool was applied.

### Meta-analysis

STATA 12.0 and RevMan 4.3 were used to synthesize the results.

For incidence calculations we used the incidence of events in the numerator and the evaluated population in the denominator. The incidence of individual and pooled studies was estimated using the Freeman-Turkey transformation (double arcsine transformation) to adjust the limiting the CI among 0-100%.[9, 10] For the comparison of intramuscular route vs. others, we used a Mantel-Haenszel method to pool the data using risk ratios (RRs).

The random effects model was used by default. If studies reported to have zero events, we applied a correction factor of 0.5 to allow for the inclusion of those studies in the analysis.[11] Statistical heterogeneity was assessed using I^2^, which describes the percentage of the variabilitythat is attributable to heterogeneity rather than chance.[12] Publication bias was assessed through the Egger test [13].

## Results

**Fig. 1:**
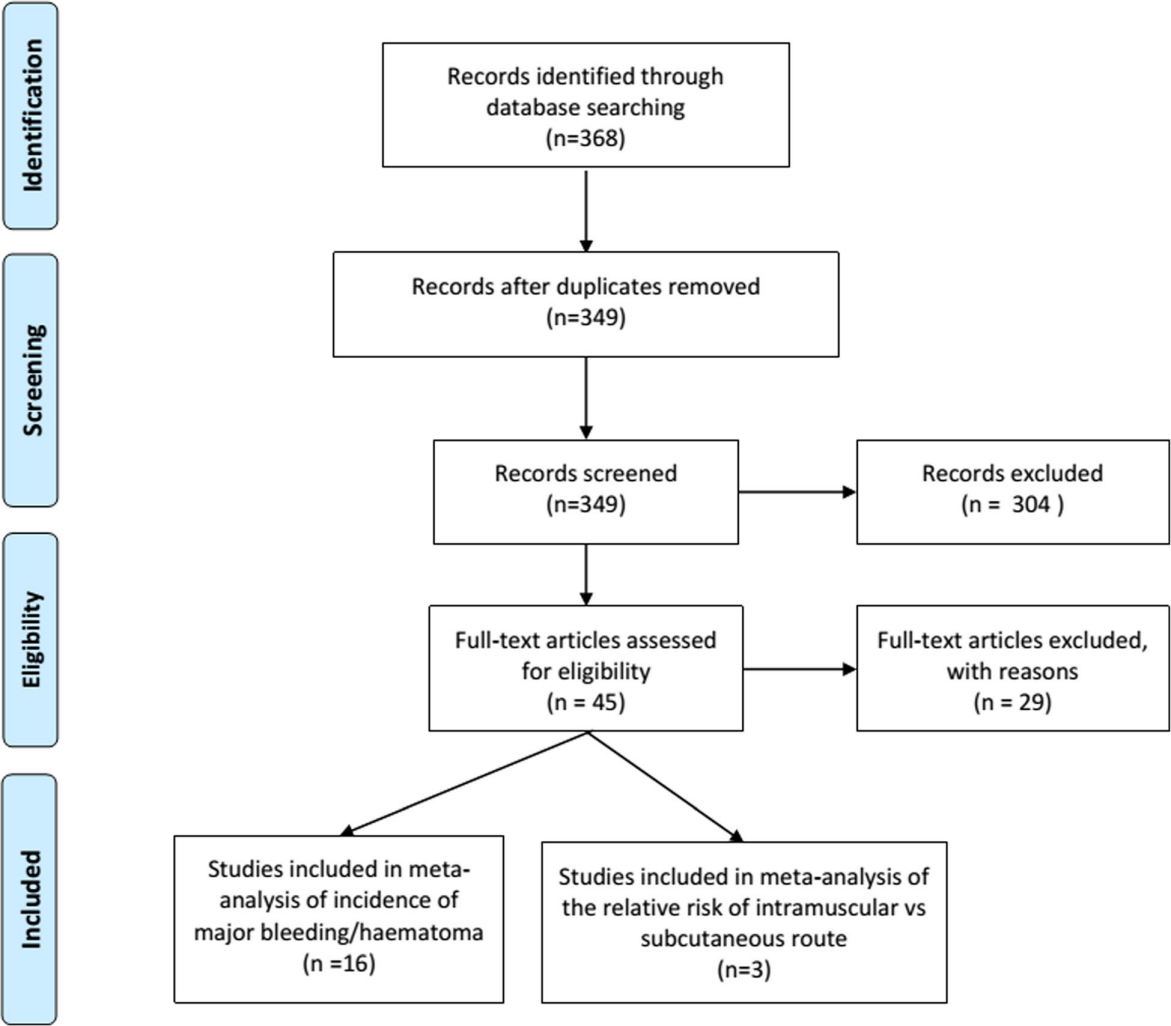
Flowchart of studies selection

The study search yielded 368 records, from which 16 fulfilled the inclusion criteria. All the 16 studies had data (including zero events) used for the estimation of bleeding events in patients anticoagulated submitted to vaccination[14–29], and 3 studies had comparative data about the risks of intramuscular route vs. subcutaneous route [22, 24, 26] (Fig. 1). Influenza vaccination was the commonest vaccine in the included studies. Most of the studies included only patients treated with VKA. The main characteristics of the included studies are shown in Table 1.

**Table 1:** Studies included in the review

Study	Sample size	Mean/ median age	Anticoagulant	Indication for anticoagulation	Vaccine used and route(s) of administration	Follow-up
Patriarca 1983	33	Not reported	Warfarin	Not reported	Influenza vaccination Route not reported	30 days
Lipsky 1984	21	62.5 years	Warfarin	Not reported	Influenza vaccine Route not reported	28-30 days
Kramer 1984	8	Not reported	Warfarin	Not reported	Influenza vaccine Route not reported	21 days
Gomolin 1985	15	Not specified (geriatric)	Warfarin	Not reported	Influenza vaccine Route not reported	21 days
Weibert 1986	13	N/R	Warfarin	Not reported	Influenza vaccine Route not reported	14 days
Bussey 1988	24	60.3 years	Warfarin	Not reported	Influenza vaccine Route not reported	, 4 months
Arnold 1990	9	68 years	Warfarin	Not reported	Influenza vaccine Route not reported	30 days
Raj 1995	41	65.7 years	Warfarin	Not reported	Influenza vaccine Route: im	14 days
Delafuente 1998	36	68 years	Warfarin	Not reported	Influenza vaccine Route: im vs. sc	4 months
Paliani 2003	90	74 years	Warfarin (98%), acenocoumarol (2%)	Not reported	Influenza vaccine Route: im	7-10 days
Ballester Torrens 2005	59	72.4 years	Not specified	Atrial fibrillation (majority), valvular prosthesis (10%)	Influenza vaccine Route: im vs. sc	7 months
MacCallum 2007	106 (INR analysis only 78)	73.7 years	Warfarin	Not reported	Influenza vaccination Route not known, possibly im	3 months
Casajuana 2008	229	73.6 years	Acenocoumarol (98%), warfarin (2%)	Atrial fibrillation (70%), valvular heart disease (17%), ischemic heart disease (12%)	Influenza vaccine. Route: im vs. sc	10 days
Iorio 2010	104	71.3 years	Warfarin	Atrial fibrillation (54%), venous tromboembolism (14%), aortic valve prosthesis (12%), dilated cardiomyopathy (12%), mitral valve prosthesis (6%), mitral and aortic valve prosthesis (2%)	Influenza vaccine Route: im	28 days
Van Aalsburg 2011	19 (im) 9 (sc)	65 years (im) 57 years (sc)	89% oral anticoagulants, 11% combination platelet anti-aggregate therapy	Not reported	DTP, HepA, Hib, typhoid fever vaccine, combination of HepA and HepB Route: im HepA Route: sc	3 days
Bauman 2016	28	5 years	Warfarin	Congenital heart disease (86%), Kawasaki syndrome (7%), others	Influenza vaccine (82%), combinations of PCV, DTaP-IPV, MMR, MMRV, MenC, Hib, HepA, HepB, palivizumab Route: im, sc	6 days (1-14 days)

*BCR* British Corrected Ratio, *DOACs* direct oral anticoagulants, *DTP* diphtheria, tetanus and polio virus vaccine, *DTaP-IPV* diphtheria, tetanus, acellular pertussis, inactivated polio virus combination vaccine, *Hib* Hemophilus influenzae type B vaccine, *HepA* hepatitis A vaccine, *HepB* hepatitis B vaccine, *im* intramuscular, *INR* International Normalized Ratio, *MenC* conjugate meningococcal type C vaccine, *MMR* measles, mumps and rubella vaccine, *MMRV* measles, mumps rubella and varicella vaccine, *PCV* pneumococcal conjugate vaccine, *sc* subcutaneous

### Risk of bias

The risk of bias of all studies was classified as moderate with score ranging between 5 and 6 (Supplementary Table 3).The major sources of bias are related to the small sample sizes, and the definition of the exposure which as deemed to be intramuscular due to the type of vaccine used in the older studies. Regarding the 3 RCTs included, the most remarkable feature of risk of bias, in particular performance bias, was the single-blinded nature of all trials (Supplementary Table 4).

### Incidence of major bleeding or haematomas

**Fig. 2:**
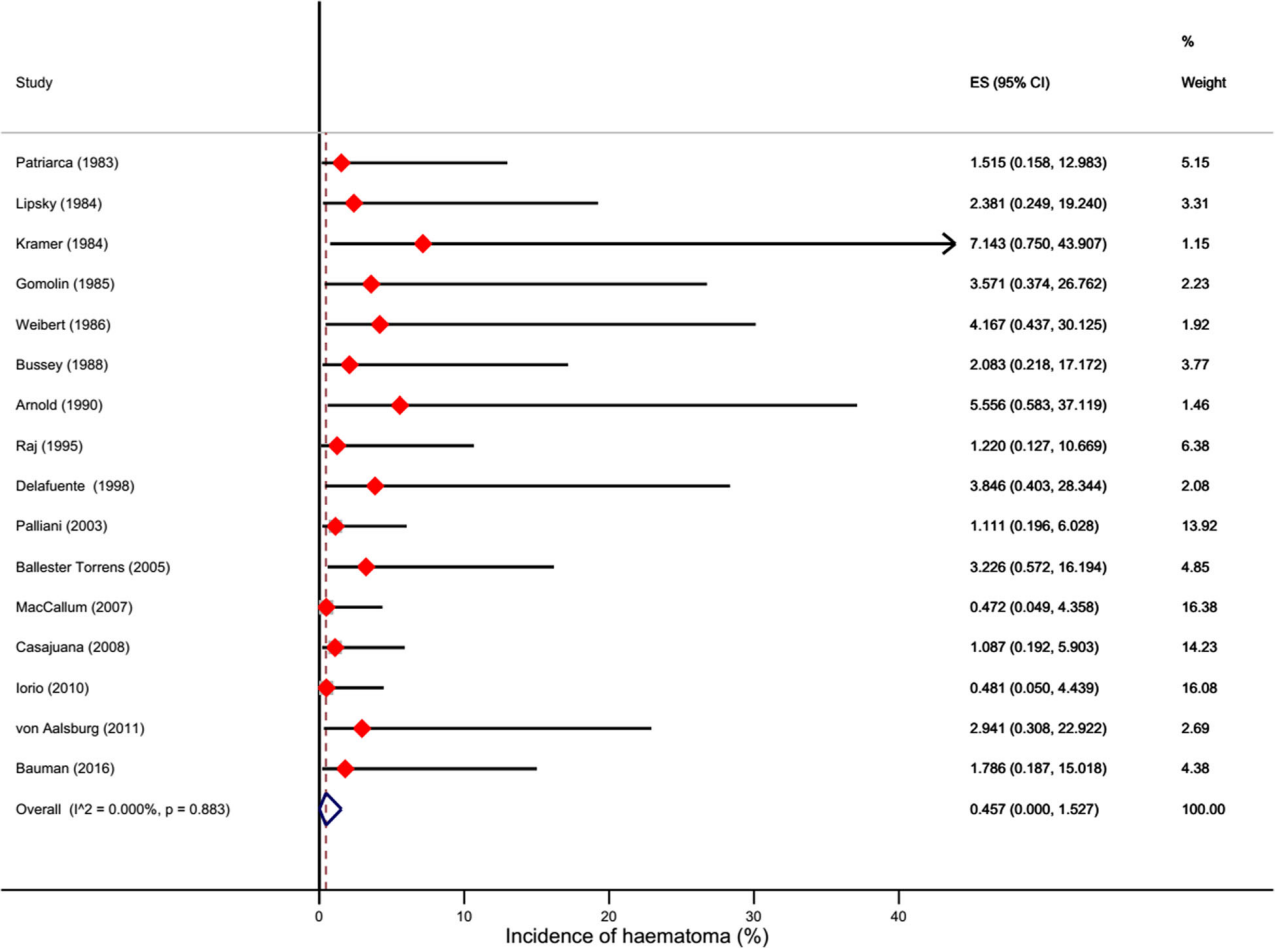
Forest plot showing the incidence of haematomas

Among the included studies, no major bleeding was reported. The pooled data of 16 studies enrolling 642 anticoagulated patients showed that the estimated incidence of haematomas was 0.46% (95%CI 0-1.53%) (Fig. 2). There was no significant heterogeneity (I^2^=0%).

Exploratory analyses showed that when only studies at lower risk bias were included the incidence was 0.44% (95%CI 0-1.60%). This incidence was lower but not significantly so than that estimated for higher risk of bias studies (1.80%, 95%CI 0-5.75) (Supplementary Fig. 1; Table 2). Also, different methods to handle zero events did not show substantial changes in the estimates (Table 2; Supplementary Figs. 2 and 3).

**Table 2:** Results of subgroup/exploratory analyses

Subgroup/Method	Incidence (%)	95% Confidence interval	I^2^
Moderate-Low risk of bias	0.44	0.00-1.60	0%
Moderate-High risk of bias	1.80	0.01-5.75	0%
Adding 0.5 to zero cells (primary approach)	0.46	0.00-1.53	0%
Adding 0.1 to zero cells	0.03	0.00-0.65	0%
No addition	<0.001	0.00-0.25	0%

The Egger test was performed to raw data (i.e. without continuity correction) and it did not suggest publication bias (Supplementary Fig. 4).

### Intramuscular vs. subcutaneous route for vaccination

**Fig. 3:**
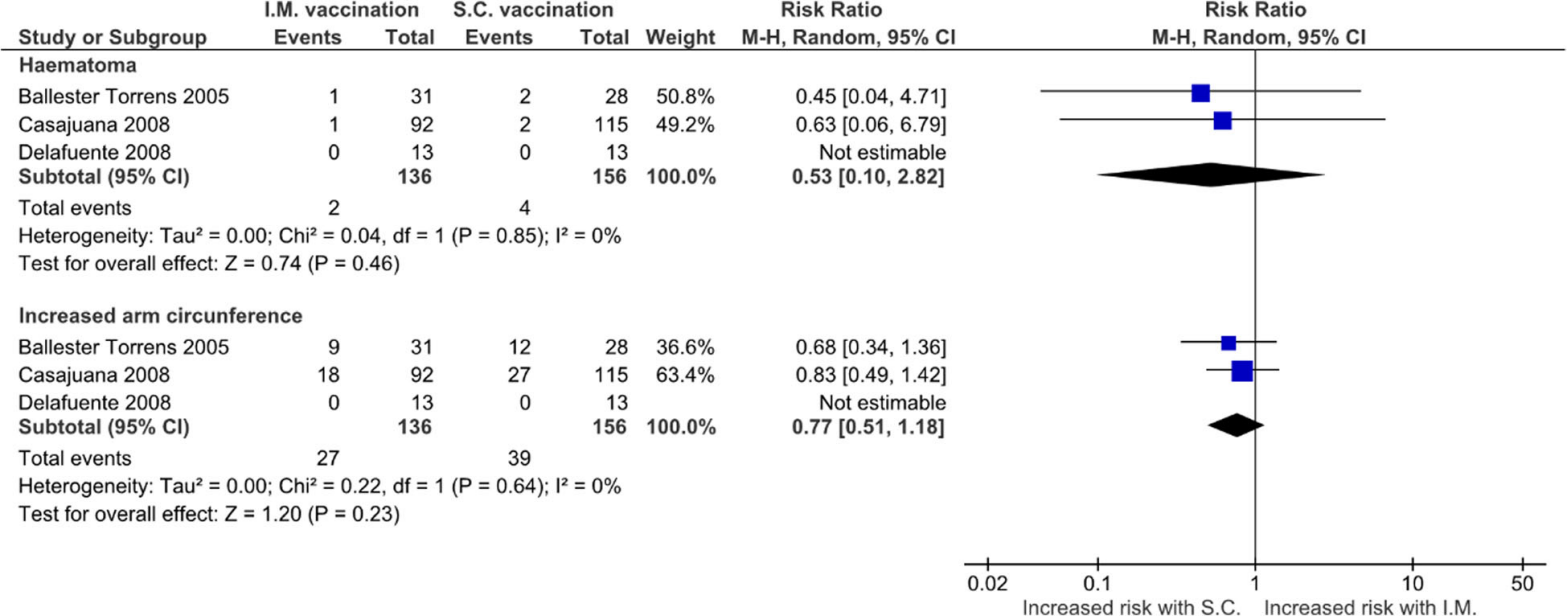
Forest plot showing the comparison of the risks of intramuscular vaccination and subcutaneous vaccination in anticoagulated patients

There were 3 studies reporting data about haematomas for intramuscular and subcutaneous route. Intramuscular route did not increase the risk of haematoma (RR 0.53, 95%CI 0.10-2.82; I^2^=0%; 2 studies, 266 patients) nor the risk of increased arm circumference (RR 0.77, 95%CI 0.51-1.18; I^2^=0%; 2 studies, 266 patients) (Fig. 3). Publication bias was not formally evaluated due to the small number of studies (Supplementary Fig. 5).

## Discussion

The main results of our systematic review were: (1) There was no report of major bleeding events related with intramuscular vaccination; (2) The incidence of haematomas was very low among these patients treated with oral anticoagulants; (3) The comparative risk of haematomas through intramuscular vaccination is not higher than the subcutaneous route in patients treated with oral anticoagulants.

These results are particularly relevant for patients treated with oral anticoagulants because they are usually at high risk of cardiovascular events due to their baseline diagnosis. This high-risk group can be considered also to be at high risk of bleeding complications, and caution with previous risk/benefits ascertainments were recommended[30]. However, vaccines seem trend towards the disease prevention supporting the benefit[31–33], and our results support the absence of substantial bleeding risk. In fact, Influenza vaccination is recommended for patients with coronary disease and heart failure[34], important risk factors for atrial fibrillation, which is the most prevalent cause for needing chronic oral anticoagulation. The relevance of this topic increases with the vaccination for COVID-19 because patients with atrial fibrillation are at high-risk of mortality[35], and most of these patients are at high-risk for complications for COVID-19 and belong to priority groups.

The “COVID-19: the green book” is a British document that has guidance for vaccination anticoagulated patients.[36] It is important to mention that this book states that there are very few individuals who cannot receive the vaccines. As for care regarding patients on stable anticoagulation therapy, supratherapeutic treatment should be avoided (by confirming non-supratherapeutic International Normalised Ratio – INR in the last measure) and a fine needle (23 or 25 gauge) should be used for the vaccination, followed by firm pressure applied to the site without rubbing for at least 2 min.

These precautions are overall shared in intramuscular procedures such as the administration of botulinum toxin in neurological conditions,[37] without any safety warning. In other conditions requiring intramuscular injections, such as the administration of penicillin in patients treated with oral anticoagulants, data has shown to be safe with a low incidence of haematomas.[38].

The subcutaneous route has been considered as a possible strategy to avoid bleeding complications of vaccination in anticoagulated patients. Besides the potential problems of inadequate immunoreactivity/vaccine efficacy[39], this route did not show increased safety. In fact, in one study the subcutaneous route showed increased risk of cutaneous lesions and higher values in pain scales at 24 h.[26].

Our results are limited by the small sample sizes of the studies included. Larger population-based studies would be necessary to determine the prevalence of major bleeding events and haematomas related to intramuscular vaccination, which seems to be a rare event. The safety concerns and strict monitoring of COVID-19 vaccination could be an interesting opportunity to collect and report those data. Some studies were included deeming that the vaccination was intramuscular, however this option showed to be conservative because the studies at lower risk of bias had lower incidences of haematoma. Vitamin K antagonist are still recommended for few clinical entities, such as mechanical prosthetic heart valve or significant mitral stenosis but, nowadays, an important share of anticoagulated patients is treated with DOACs [40, 41], which were not represented in our review. Nevertheless, DOACs seem to be safer that warfarin in terms of bleeding,[42] and we cannot exclude some interaction between the vaccine and the INR in patients receiving warfarin despite many studies stating against it[25, 27, 43]. Overall, these limitations suggest that our results can be less frequent than our estimates, stressing the safety of intramuscular vaccination in this population.

## Conclusions

Intramuscular vaccination in anticoagulated patients is safe, with a very low incidence of haematomas. The best available evidence suggests that using the intramuscular route does not increase the risk of haematomas compared with the subcutaneous route. Anticoagulated patients and healthcare personnel involved in vaccination should be reassured regarding intramuscular vaccinations.

## Supplementary information

**Additional file 1**

## Data Availability

Not applicable.
