# Supplementary material for: Low risk of haematomas with intramuscular vaccines in anticoagulated patients: a systematic review with meta-analysis
Source: Thromb J. 2022 Feb 16;20:9. doi: 10.1186/s12959-022-00367-1 (PMC8848629; doi:10.1186/s12959-022-00367-1)
Supplement: Supplementary file 1 — Additional file 1 [file 12959_2022_367_MOESM1_ESM.pdf]

SUPPLEMENTARY MATERIAL

Low risk of haematomas with intramuscular vaccines in anticoagulated patients: a systematic review with meta-analysis

Authors: Daniel Caldeira, Bárbara Sucena Rodrigues, Mariana Alves, Fausto J. Pinto, Joaquim J Ferreira.

Corresponding author:
Daniel Caldeira, MD, PhD
Laboratory of Clinical Pharmacology and Therapeutics, Faculdade de Medicina, Universidade de Lisboa, Portugal. Centro Cardiovascular da Universidade de Lisboa - CCUL, CAML, Faculdade de Medicina, Universidade de Lisboa, Portugal.

Contents

Supplementary Table 1: Search Strategy 3

Supplementary Table 2: Key excluded studies 4

Supplementary Table 3: Risk of bias evaluation using an adapted tool of Hoy et al. 5

Supplementary Table 4: Risk of bias evaluation using the Cochrane Risk of Bias Tool 6

Supplementary Figure 1: Incidence of haematomas according with the risk of bias. 7

Supplementary Figure 3: Forest plot evaluating the incidence of haematomas without the continuity correction. 8

Supplementary Figure 4: Regression line representing the Egger test. 8

Supplementary Figure 5: Funnel plot for the studies and estimates included in the intramuscular vs. subcutaneous comparison. 9

Supplementary Table 1: Search Strategy

#	Searches
1	vaccin*.ti,ab.
2	exp Vaccines/
3	exp Immunization/
4	immunization.ti,ab.
5	1 or 2 or 3 or 4
6	anticoagulat*.ti,ab.
7	anticoagulant*.ti,ab.
8	hypocoagula*.ti,ab.
9	exp Anticoagulants/
10	warfarin.ti,ab.
11	acenocoumarol.ti,ab.
12	phenprocoumon.ti,ab.
13	fluindione.ti,ab.
14	NOAC*.ti,ab.
15	non-vitamin K antagonist oral anticoagulant*.ti,ab.
16	DOAC*.ti,ab.
17	direct oral anticoagulant*.ti,ab.
18	apixaban.ti,ab.
19	dabigatran.ti,ab.
20	edoxaban.ti,ab.
21	rivaroxaban.ti,ab.
22	6 or 7 or 8 or 9 or 10 or 11 or 12 or 13 or 14 or 15 or 16 or 17 or 18 or 19 or 20 or 21
23	5 and 22

Supplementary Table 2: Key excluded studies

**Study (author and year)**	**Reason for exclusion**
Ionescu 1984	Wrong publication type
DeDea 2011	Wrong publication type
DeDea 2012	Wrong publication type
Behring 2009	Wrong drug
Mancano 2018	Wrong publication type
Marder 2005	Wrong publication type
Iorio 2005	Incomplete version of included study
Casajuana 2005	Incomplete version of included study
Porter 2011	Wrong publication type
Ringwald 2009	Wrong publication type
Schwartz 2009	Wrong publication type
Sharma 2007	Wrong publication type
Sigell 1970	Wrong publication type
Thompson 2016	Wrong publication type
Yang 2014	Wrong publication type
Gomolin 1986	Incomplete version of included study
Jackson 2007	Wrong outcome
Poli 2002	Wrong intervention
Chiarugi 1999	Wrong intervention
Wiedermann 2012	Wrong population
Deibe 2008	Ongoing study
Wartel 2020	Wrong population

Supplementary Table 3: Risk of bias evaluation using an adapted tool of Hoy et al.

Studies	Representation	Sampling	Random selection	Minimal non-response	Direct data collection	Acceptable case definition	Measured parameter	Data collection mode	Numerator and denominatior adequate	Summary overall risk
Patriarca 1983										Moderate
Lipsky 1984										Moderate
Kramer 1984										Moderate
Gomolin 1985										Moderate
Weibert 1986										Moderate
Bussey 1988										Moderate
Arnold 1990										Moderate
Raj 1995										Moderate
Delafuente 1998										Moderate
Palliani 2003										Moderate
Ballester Torrens 2005										Moderate
MacCallum 2007										Moderate
Casajuana 2008										Moderate
Iorio 2010										Moderate
von Aalsburg 2011										Moderate
Bauman 2016										Moderate

Supplementary Table 4: Risk of bias evaluation using the Cochrane Risk of Bias Tool

	Random sequence generation (selection bias)	Allocation concealment (selection bias)	Blinding of participants and personnel (performance bias)	Blinding of outcome assessment (detection bias)	Incomplete outcome data (attrition bias)	Selective reporting (reporting bias)	Other bias
Ballester Torrens 2005	+	+	−	?	+	+	+
Casajuana 2008	+	+	−	?	+	+	+
Delafuente 1998	?	?	−	?	?	−	+

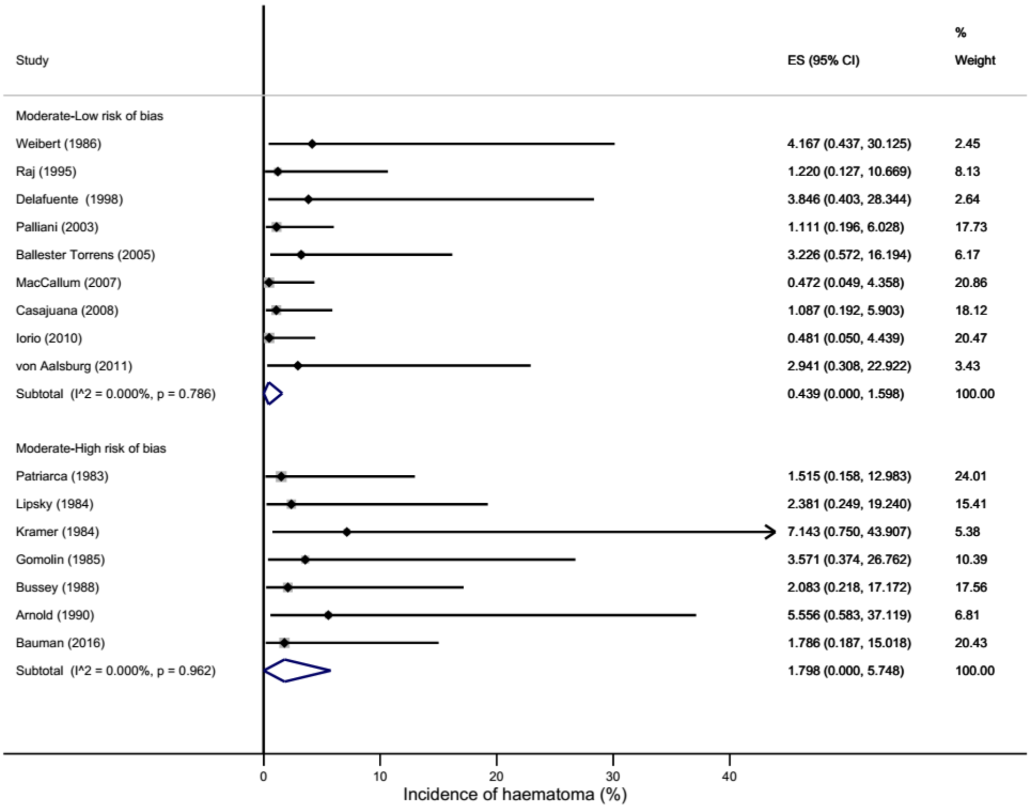

Supplementary Figure 1: Incidence of haematomas according with the risk of bias.

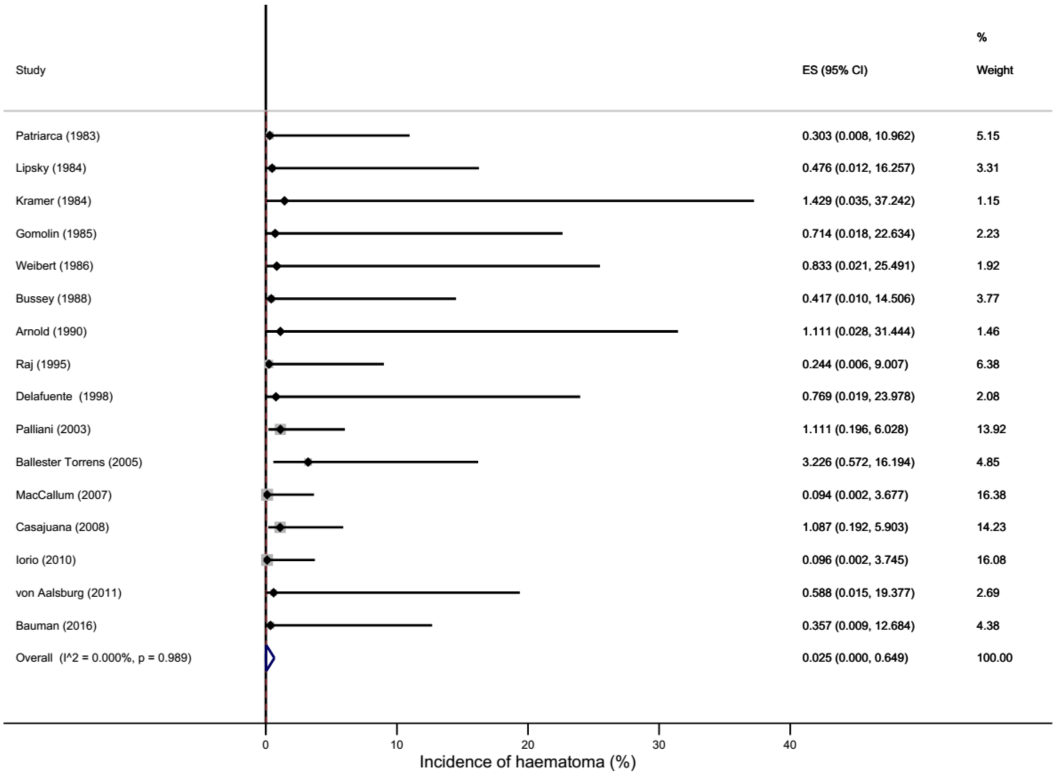

Supplementary Figure 2: Forest plot evaluating the incidence of haematomas using the continuity correction of 0.1, when required.

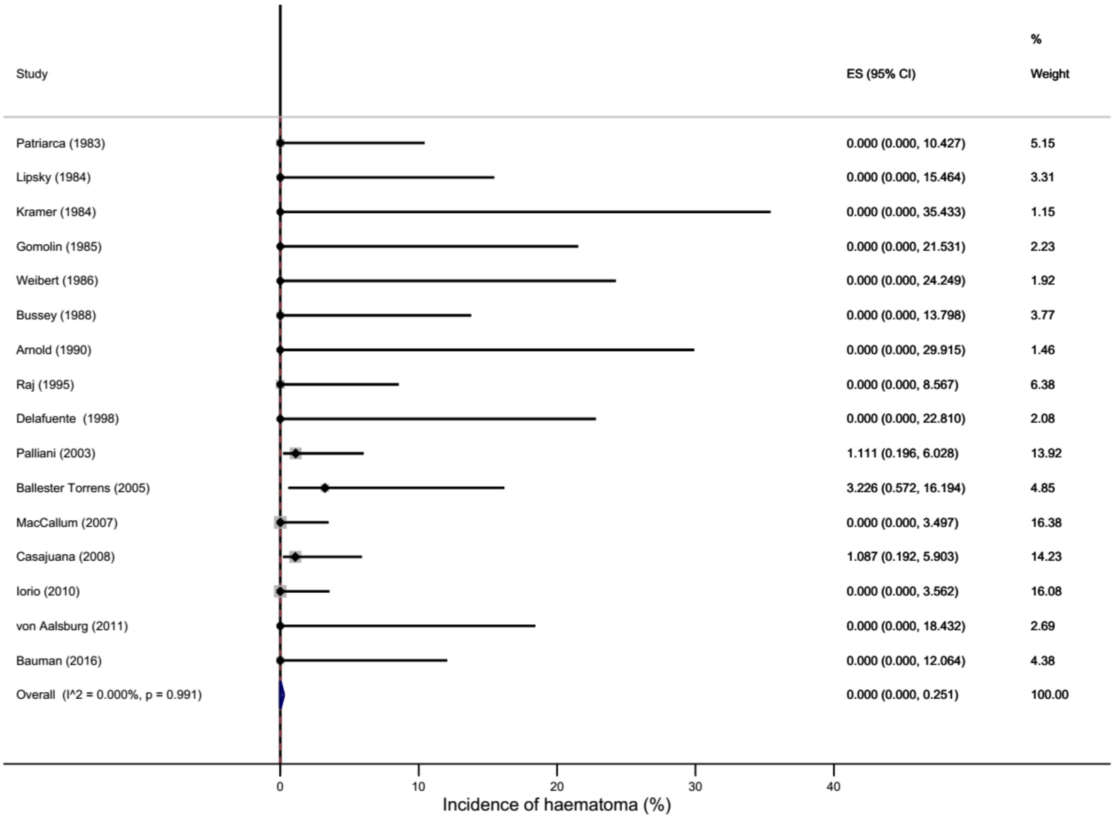

Supplementary Figure 3: Forest plot evaluating the incidence of haematomas without the continuity correction.

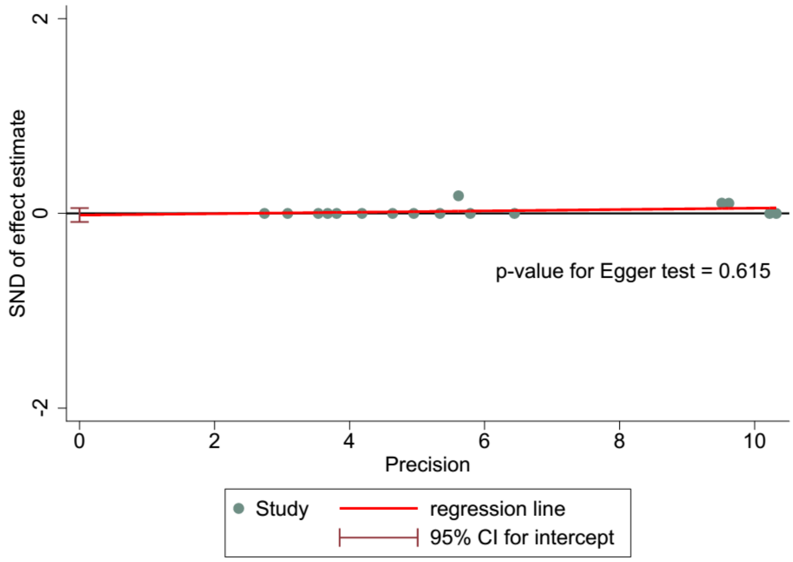

Supplementary Figure 4: Regression line representing the Egger test.

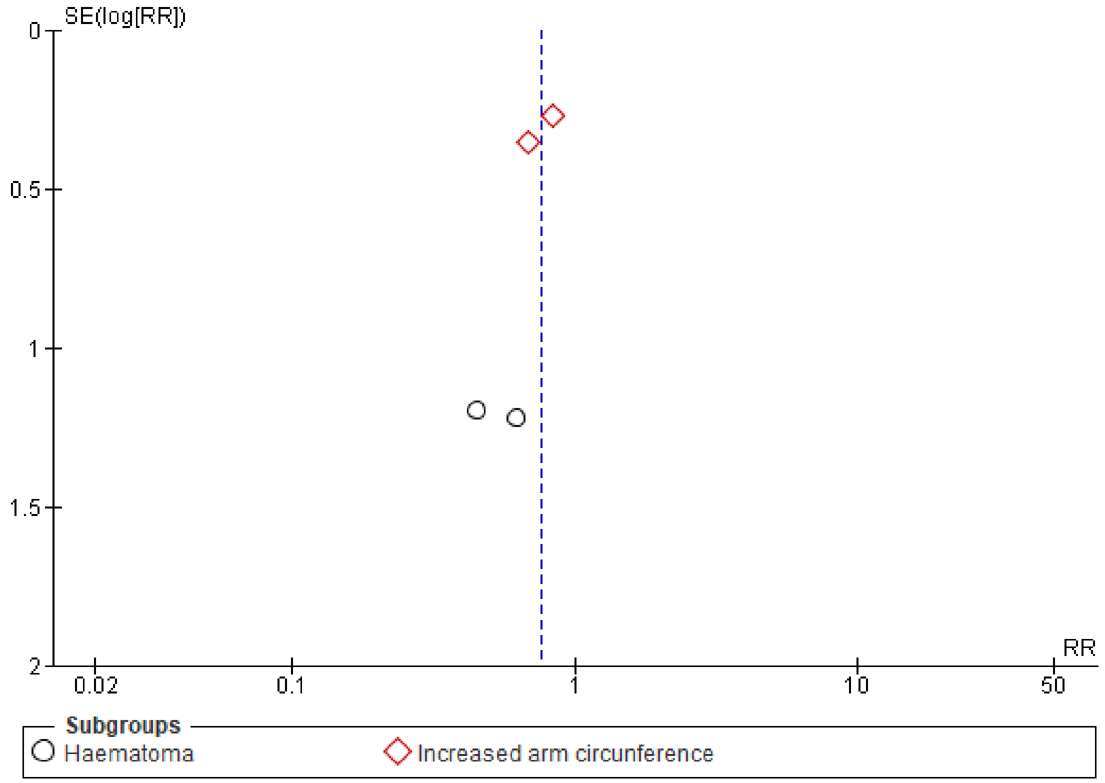

Supplementary Figure 5: Funnel plot for the studies and estimates included in the intramuscular vs. subcutaneous comparison. Due to the small number of studies, formal publication bias is not recommended.
